# The complete mitogenome of the Moroccan *Luciobarbus rifensis* Doadrio, Casal-López & Yahyaoui, 2015 (Actinopterygii: Cyprinidae)

**DOI:** 10.1080/23802359.2016.1250137

**Published:** 2017-01-05

**Authors:** Diushi Keri Corona-Santiago, Miriam Casal-López, Silvia Perea, Ignacio Doadrio

**Affiliations:** Departamento de Biodiversidad y Biología Evolutiva, Museo Nacional de Ciencias Naturales, CSIC, Madrid, España

**Keywords:** Rifian barbel, cyprinid, mitogenome, North Africa

## Abstract

The Rifian barbel (*Luciobarbus rifensis*) is a tetraploid cyprinid species from North Africa. The aim of this work is to characterize the mitogenome of *Luciobarbus rifensis* in order to contribute in the future exploration of regions. The circular mitogenome of the Rifian barbel (16 607bp) consists of 37 genes (13 protein-coding genes, 2 rRNA genes, and 22 tRNA genes). The cyprinid mitogenome is most related to *L. rifensis* available is *L. capito*.

The species *Luciobarbus rifensis* Doadrio, Casal-López, and Yahyaoui, 2015 is representative of the fish family Cyprinidae and endemic of rivers from the Rif area of Morocco (Casal-López et al. 2015). The molecular marker discovered is relevant due the tetraploid condition in *Luciobarbus*. The aim of this work was to characterize the mitochondrial genome of *L. rifensis* to contribute to the future exploration of novel regions that will be apply to genetic and evolution studies in this fish group.

We used a tissue (caudal fin) of *Luciobarbus rifensis* collected in the Laou River (Mediterranean slope from Morocco) in Chefchaouen province (35°07′08.2”N 5°17′20.7”W), stored in the fish collection of the National Museum of Natural Sciences of Madrid, Spain (Voucher specimen: MNCN-235026). Library of genomic-DNA was prepared, tagged and subjected for sequencing in the Illumina MiSeq platform (PE300) in AllGenetics & Biology, SL. Assemblage was performed using SOAPdenovo2 (Luo et al. 2012) and quality evaluation using FastQC (Andrews 2010). Identification of tRNA genes was estimated using tRNAscanSE v1.21 (Schattner et al. 2005). Annotation confirmation was implemented using MitoAnnotator (Iwasaki et al. 2013). Phylogenetic reconstruction was performed under Neighbour-Joining assumption; including 23 species of the Cyprinidae family available on GenBank. The analysis was conducted with a full alignment built in MAFFT v7.222 (Katoh et al. 2002).

The base composition of the circular mitogenome of the *Luciobarbus rifensis* (GenBank accession: KX348041) was as follows: A = 30.9%, C = 27.9%, G = 16.8%, and T = 24.5% (GC-rich = 44.6%), consisting of 37 genes in 16 607bp (13 protein-coding genes, 2 rRNA genes, and 22 tRNA genes). The size of the tRNA genes in mitogenome is 67–77bp. The phylogenetic relationships for the species (Figure 1) were consistent with those obtained for Cyprinidae (Wang et al. 2012; Wang et al. 2013) differing in some low supported relationship (e.g. for *Gymnocypris namensis*, *Scaphiodonichthys acanthopterus,* and *Procypris rabaudi*). As it has been previously reported (Zardoya & Doadrio 1999; Casal-López et al. 2015), of the mitogenomes available, the most related with *L. rifensis* is *L. capito* followed by *Barbus barbus*. No gene rearrangement was identified in the mitogenome of *L. rifensis* with respect to the mitogenomes compared. On the light of our results, the mitogenome of *L. rifensis* will contribute to when searching new molecular markers for the study of this tetraploid fish group.

**Figure 1.**
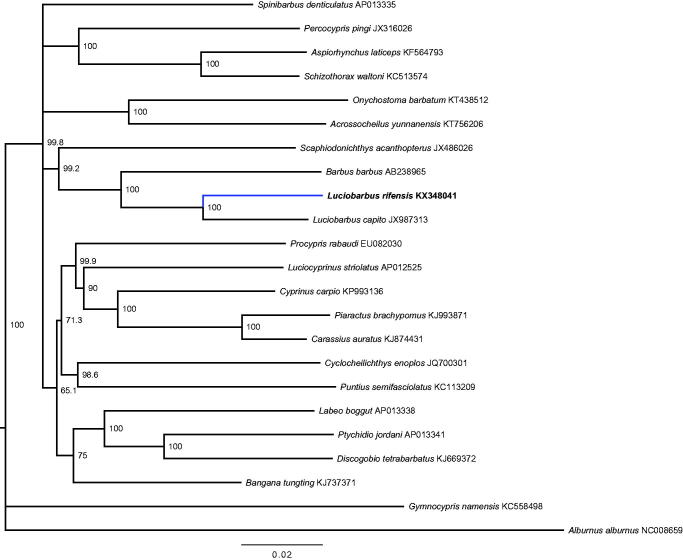
Sample validation based on Neighbour-Joining tree of 23 species of the Cyprinidae family (1000 bootstrap replicates using uncorrected *p*-distances).
